# A Rare Case of Bacterial Meningitis Caused by Hematogenous Spread of Vancomycin-Resistant *Enterococcus faecium* in an Immunocompromised Patient

**DOI:** 10.1155/2024/2193650

**Published:** 2024-07-25

**Authors:** Myeongji Kim, Christopher Graham, Ryan W. Stevens, Aditya Shah

**Affiliations:** ^1^ Division of Public Health, Infectious Diseases, and Occupational Medicine Mayo Clinic, Rochester, MN, USA; ^2^ Division of Blood and Marrow Transplant Mayo Clinic, Rochester, MN, USA; ^3^ Department of Pharmacy Mayo Clinic, Rochester, MN, USA

## Abstract

Vancomycin-resistant *Enterococcus faecium* (VRE) meningitis is rare. It is usually associated with neurosurgical procedures or devices. We describe a case of VRE meningitis from hematogenous spread during persistent bacteremia in an immunocompromised patient who received haploidentical bone marrow transplant for VEXAS syndrome. The bacteremia and meningitis were successfully treated with combination of intravenous (IV) daptomycin, ceftaroline, and linezolid.

## 1. Introduction

VRE meningitis is rare and accounts for 0.3–4.0% of all bacterial meningitis [1–5]. In patients with hematopoietic stem cell transplant, VRE infection rates have ranged from 1.4–25% [6–10]; however, the rate of VRE infections involving the central nervous system (CNS) in hematopoietic stem cell transplant patients is unknown. VRE meningitis is usually associated with neurosurgical interventions and devices (e.g., ventriculoperitoneal shunt) [11–15]. Given the limited availability of antimicrobials with activity against VRE and difficulty in assuring adequate CNS penetration of these agents, the optimal treatment of VRE meningitis remains largely unknown. We describe a case of VRE meningitis in a patient who received haploidentical bone marrow transplant for VEXAS syndrome. VEXAS syndrome is an autoinflammatory disease caused by mutations in the E1 ubiquitin-activating enzyme leading to ineffective clearance of cell waste products. It is diagnosed by identification of vacuoles in the erythrocyte and myeloid precursors on bone marrow biopsy, a UBA1 somatic mutation, and autoinflammation [16]. First line treatment involves impedance of the inflammatory response with corticosteroids or Janus-activating kinase (JAK) inhibitors. For patients who fail immunosuppression or have associated cytopenia, allogeneic stem cell transplant is a potentially curative second line option with high rates of success [16,17]. In the largest VEXAS registry, patients were found to be at risk of death at 37% in 5 years due to serious infection in up to 50% of cases; 16% of cases occurred in patients who did not receive immunosuppression, likely due to immune dysregulation [18]. Patients receiving haploidentical stem cell transplants are at higher risk for infectious complications due to post-transplant cyclophosphamide, with both invasive molds, bacterial infection, and CMV reactivation leading to 6% infection-related death [19]. We describe a case in which a patient, who received allogeneic bone marrow transplant for VEXAS syndrome, developed VRE bacteremia likely from perirectal mucosal tear leading to hematogenous seeding of the cerebrospinal fluid (CSF) and meningitis despite receiving high dose daptomycin therapy.

## 2. Case Presentation

A 72-year-old male with a history of VEXAS syndrome received conditioning chemotherapy with fludarabine, busulfan, and thiotepa, followed by haploidentical bone marrow transplant (hospital day 1) due to steroid refractory autoinflammatory disease. His previous treatment for VEXAS syndrome consisted of prednisone doses between 50 mg and 60 mg per day for over three months prior to transplant. He received cyclophosphamide on days 3 and 4 after transplant, with tacrolimus and mycophenolate mofetil starting on hospital day 5 for prophylaxis of graft-versus-host disease (GVHD) and was continued on chronic high-dose prednisone of 0.5 mg/kg (40 mg oral daily). On hospital day 15, the patient developed neutropenic fever (ANC <0.01 × 10^9^/L) with body temperature of 38.9°C. The fever was accompanied by persistent diarrhea resulting in perirectal mucosal tear. A computed tomography (CT) scan of the abdomen and pelvis with IV contrast was unrevealing for other intra-abdominal infectious source. Blood cultures revealed VRE from two sets collected from both the tunneled central catheter and peripheral venipuncture in 7 hours and 9 hours, respectively (Table 1).

**Figure 1:**
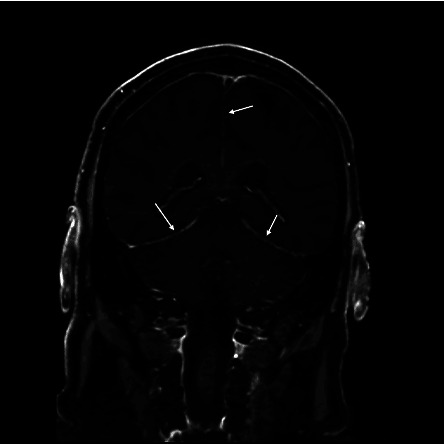
Magnetic resonance image of the brain with intravenous contrast. Postgadolinium T1 with fat saturation series showing diffuse dural thickening and enhancement along the bilateral cerebral hemispheres (arrows).

Daptomycin was promptly initiated at the dose of 10 mg/kg adjusted body weight (900 mg) IV every 24 hours. The tunneled central catheter was removed on hospital day 18 (bacteremia day 3). Blood cultures from the periphery remained positive after the catheter was removed, with brisk growth in 14 hours on hospital day 19. Given ongoing high-grade VRE bacteremia, ceftaroline was added at 600 mg IV every 8 hours and the dose of daptomycin was maximized to 12 mg/kg (1050 mg) IV every 24 hours. On the same day, the patient developed headaches and confusion. A magnetic resonance imaging (MRI) of the brain with IV contrast showed diffuse dural thickening and enhancement along the bilateral cerebral hemispheres (Figure 1). A lumbar puncture performed on hospital day 20 demonstrated 48 total nucleated cells per microliter with 95% neutrophilic pleocytosis. Gram stain of the CSF was positive for Gram-positive cocci in pairs and chains. CSF testing revealed a negative meningitis/encephalitis multiplex polymerase chain reaction (PCR) panel which included *Streptococcus pneumoniae* and *Streptococcus agalactiae*. CSF cryptococcal antigen and dedicated HSV-1 and -2 PCR were also negative. Owing to concern of VRE meningitis along with the known poor CNS penetration of daptomycin and development while on therapy at 10 mg/kg, linezolid 600 mg IV every 12 hours was added. On hospital day 22, *E. faecium*, with identical antimicrobial susceptibility testing to the hospital day 15 blood cultures, grew from the CSF cultures (Table 1).

**Figure 2:**
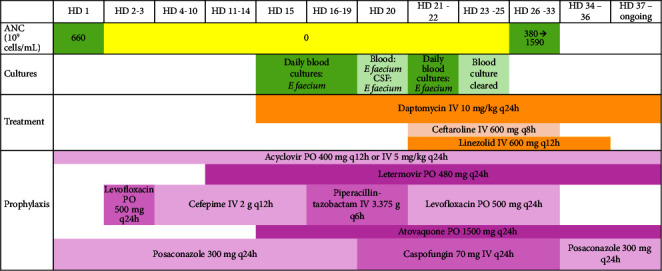
Timeline of hospital course. HD: hospital day; ANC: absolute neutrophil count; IV: intravenous; PO: oral.

Over a period of four days after linezolid initiation, he had improvement of mental status which also coincided with clearance of VRE from the bloodstream on hospital day 23. The patient was continued on triple therapy with linezolid, ceftaroline, and daptomycin. Neutrophil engraftment was noted on hospital day 26. Transesophageal echocardiography (TEE) did not demonstrate any valvular involvement. Positron emission and computed tomography (PET-CT) showed focal infection in the perineum where rectal colonization with VRE had been previously demonstrated by positive rectal swab on hospital day 18. Repeated brain MRI obtained on hospital day 32 (linezolid therapy day 13) showed resolution of dural enhancement and no other findings of intracranial infection. Also, on this day, blood cultures continued to be negative and ceftaroline was discontinued. The patient completed 2 weeks of IV linezolid, and his confusion resolved without any neurologic sequelae. Continuation of IV daptomycin for 6 weeks each from the first negative blood culture was planned given that endovascular seeding could not be ruled out in the setting of prolonged bacteremia for 8 days, and therapy is ongoing.  Figure 2 summarizes the hospital course. To date, the patient has demonstrated return of mental status to baseline and continued progression with physical rehabilitation.

## 3. Discussion

Patients with hematological or oncological conditions with or without neutropenia have high rates of VRE bacteremia and poor outcomes [6–10]. However, prevalence rates of CNS involvement with this pathogen via hematogenous seeding are unknown, and there are very few reported cases of VRE meningitis without neurosurgical intervention, especially in the immunocompromised patient population. There have been case reports of VRE meningitis after stem cell transplantation [14, 20–22] and while undergoing chemotherapy for AML [23]. We hypothesize the risk of VRE meningitis in this patient was predominantly driven by host and treatment factors as opposed to pathogenic factors, as he was on chronic high dose corticosteroids and received several lymphodepleting chemotherapies to prevent graft failure and GVHD. Haploidentical stem cell transplantation bears high risk for graft failure, GVHD, and infectious complications. Strategies to reduce this include T-cell lymphodepletion of the host and the donor stem cells [19, 24, 25]. For lymphodepletion of the host, this includes a combination of two lymphodepletion chemotherapies, which in his case was fludarabine and thiotepa [26]. In addition, lymphodepletion of the graft occurs using post-transplant cyclophosphamide which causes senescence of allo-reactive T-cells [19, 24, 25]. The addition of chronic steroid therapy in this circumstance adds to lymphodepletion of both T-cells and B-cells. Receiving such extensive lymphodepleting chemotherapy with the addition of mucositis and neutropenia due to busulfan chemotherapy likely played a role in the hematogenous seeding of VRE into the CNS in this patient.

There are no clear guidelines for treatment of CNS involvement with VRE; however, the armamentarium of agents for this organism is relatively limited to options such as linezolid and daptomycin. Daptomycin has long been utilized for VRE infections; however, elevated MICs often dictate the need for higher doses, especially in the context of severe infections. Even then, some have suggested that daptomycin demonstrates a low likelihood of pharmacodynamic target attainment of *f*AUC/MIC >27.43 mg × h/L in Monte Carlo simulations of bloodstream infections with *E. faecium* with a daptomycin MIC of 4 *μ*g/mL [27]. Some data have shown that ceftaroline (or ampicillin) coadministration with daptomycin may provide synergy through the reduction of daptomycin MICs [28]. Such combination regimens have been employed as salvage regimens in VRE bacteremia; however, data are lacking regarding efficacy of this combination in the central nervous system and are further complicated by daptomycin's poor penetration into CSF (9–11%) [29]. Furthermore, development of VRE meningitis in a patient receiving high-dose (12 mg/kg) daptomycin for VRE bloodstream infection has been previously reported [30]. Since the first case of treating VRE meningitis with linezolid was reported in 2001[31], linezolid monotherapy or as part of combination therapy with other VRE-active agents has remained a mainstay of treatment. In contrast to daptomycin, linezolid is known to have superior CSF and brain tissue penetration [32, 33]. In review of the literature, Lee et al. found total 22 cases of VRE meningitis treated with either linezolid or daptomycin from 2001 to April 2020 [34]. 15 of 19 cases treated with linezolid reported clinical cure (53.3% were monotherapy) and 5 of 9 cases treated with IV daptomycin reported cure [34]. Another strategy, intrathecal antibiotic administration, in patients who have CNS device such as external ventricular drainage or ventriculoperitoneal shunt has been suggested. Intraventricular daptomycin dosing and frequencies have been provided in healthcare-associated meningitis and ventriculitis guidelines from the Infectious Diseases Society of America [35]. Lee et al. found four cases of VRE meningitis where intrathecal or intraventricular daptomycin was utilized and achieved clearance from CSF [34]. More recently, a few antimicrobials with activity against VRE have emerged to world markets. Such agents include the lipoglycopeptide oritavancin and the tetracycline derivative eravacycline; however, these agents currently lack clinical data demonstrating efficacy in the management of VRE CNS infections and further study is warranted.

In conclusion, this case demonstrates the devastating complications of high-grade VRE bacteremia in a patient who received bone marrow transplantation as well as raises concern surrounding use of daptomycin as a therapeutic agent in this circumstance. In addition, it supports the utilization of linezolid in VRE CNS infections; however, future studies are needed to inform management of VRE meningitis in this patient population.

## Figures and Tables

**Table 1:** Antimicrobial susceptibility of *Enterococcus faecium* from blood and cerebrospinal fluid.

	MIC values (*μ*g/mL)		Interpretation
	Blood^*∗*^ (hospital day 15)	CSF (hospital day 20)	
Daptomycin	4	4	Susceptible: dose dependent
Gentamycin synergy	≤500	≤500	Susceptible
Linezolid	≤2	≤2	Susceptible
Penicillin	>8	>8	Resistant
Vancomycin	>16	>16	Resistant

MIC: minimum inhibitory concentration; CSF: cerebrospinal fluid. ^*∗*^Antimicrobial susceptibility testing performed on positive blood cultures from hospital day 22 yielded identical results.

## Data Availability

Data sharing not applicable to this article as no datasets were generated or analysed during the current study.
